# Supplementary material for: Evidence-based dust exposure prediction and/or control tools in occupational settings: A scoping review protocol
Source: PLoS One. 2024 Oct 17;19(10):e0309967. doi: 10.1371/journal.pone.0309967 (PMC11486402; doi:10.1371/journal.pone.0309967)
Supplement: S2 Appendix — (DOCX) [file pone.0309967.s002.docx]

**Appendix II: Preliminary database search Strategy**

Database: PubMed

Filters: English,

Publication date: 1000/1/1 to 23/06/2024

Search conducted: June 23, 2024

| **Search** | Terms | Retrieved records |
| --- | --- | --- |
| **#1** | (("Dust" [Mesh] OR Dust[tiab] OR silicon dioxide[tiab] OR Particulate Matter[tiab] OR Silica[tiab] OR Quartz [tiab] OR metal [tiab] OR Aerosol[tiab] OR Coal[tiab] OR Organic[tiab] OR inorganic [tiab] OR mineral dust [tiab] OR wood [tiab] ) ) AND ((Dust OR silicon dioxide OR Particulate Matter OR Silica OR Quartz OR metal dust OR Aerosol dust OR Coal dust OR Organic dust OR inorganic dust OR mineral dust OR wood dust)) | [178,768](https://pubmed.ncbi.nlm.nih.gov/?term=%28%28%22Dust%22+%5BMesh%5D+OR+Dust%5Btiab%5D++OR+silicon+dioxide%5Btiab%5D++OR+Particulate+Matter%5Btiab%5D++OR+Silica%5Btiab%5D++OR+Quartz+%5Btiab%5D+OR+metal+%5Btiab%5D++OR+Aerosol%5Btiab%5D+OR+Coal%5Btiab%5D+OR+Organic%5Btiab%5D+++OR+inorganic+%5Btiab%5D++OR+mineral+dust+%5Btiab%5D+OR+wood+%5Btiab%5D+%29+%29+AND+%28%28Dust+OR+silicon+dioxide+OR+Particulate+Matter+OR+Silica+OR+Quartz+OR+metal+dust+OR+Aerosol+dust+OR+Coal+dust+OR+Organic+dust+OR+inorganic+dust+OR+mineral+dust+OR+wood+dust%29%29&sort=&filter=dates.1000%2F1%2F1-2024%2F3%2F20&filter=lang.english) |
| **#2** | Web-based tools[tiab] OR Tools[tiab] OR "Methods"[Mesh] OR Methods [tiab] OR assessment tool[tiab] OR technology[tiab] OR silica control tool[tiab] OR REACH tool[tiab] OR ECETOC[tiab] OR TRA[tiab] OR ART[tiab] OR TREXMO[tiab] OR Stoffenmanager[tiab] OR Advanced REACH Tool (ART) [tiab] OR MEASE[tiab] OR EMKG-Expo-Tool [tiab] | [7,137,561](https://pubmed.ncbi.nlm.nih.gov/?term=Web-based+tools%5Btiab%5D+OR+Tools%5Btiab%5D+OR+%22Methods%22%5BMesh%5D+OR+Methods+%5Btiab%5D+OR+assessment+tool%5Btiab%5D+OR+technology%5Btiab%5D+OR+silica+control+tool%5Btiab%5D+OR+REACH+tool%5Btiab%5D+OR+ECETOC+TRA%5Btiab%5D+OR+ART%5Btiab%5D+OR+TREXMO%5Btiab%5D+OR+Stoffenmanager%5Btiab%5D+OR+Advanced+REACH+Tool+%28ART%29%5Btiab%5D+OR+MEASE%5Btiab%5D+OR+EMKG-Expo-Tool%5Btiab%5D&sort=&filter=dates.1000%2F1%2F1-2024%2F3%2F20&filter=lang.english) |
| **#3** | "Risk Assessment"[Mesh] OR Risk Assessment [tiab] OR Exposure assessment[tiab] OR "Occupational Exposure"[Mesh] OR Occupational Exposure[tiab] OR occupational exposure assessment[tiab] OR occupational exposure model[tiab] Exposure model[tiab] OR Exposure model assessment[tiab] OR exposure measurement[tiab] OR exposure scenarios[tiab] OR control banding[tiab] OR risk assessment[tiab] OR Risk management[tiab] OR exposure control[tiab] OR exposure control plan[tiab] | 111,295 |
| **#4** | #1AND #2 AND #3 | 890 |
