# Supplementary material for: Evidence-based dust exposure prediction and/or control tools in occupational settings: A scoping review protocol
Source: PLoS One. 2024 Oct 17;19(10):e0309967. doi: 10.1371/journal.pone.0309967 (PMC11486402; doi:10.1371/journal.pone.0309967)
Supplement: S3 Appendix — (DOCX) [file pone.0309967.s003.docx]

**Appendix III****: Search strategy for Google Advanced Search logic grid**

| **Dust** | **Evidence-based tools** |  |
| --- | --- | --- |
| (“dust*” OR particulate matter OR “particle*” OR organic* OR “metal*” OR mineral* OR inorganic OR "airborne dust") | (“web-based tool*” OR “occupational exposure” OR “exposure model” OR "risk assessment*"  OR "risk management*"  OR "exposure*"  OR "control band*"  OR "evaluation methods*") |  |
