# Supplementary material for: Evidence-based dust exposure prediction and/or control tools in occupational settings: A scoping review protocol
Source: PLoS One. 2024 Oct 17;19(10):e0309967. doi: 10.1371/journal.pone.0309967 (PMC11486402; doi:10.1371/journal.pone.0309967)
Supplement: S4 Appendix — (DOCX) [file pone.0309967.s004.docx]

**Appendix IV: Data extraction tool**

| Title | Author  (year) | Country  /Origin | Type/s of dust | Control banding | Type/s of Exposure Modelling | Input parameters | Accessibility | Platforms  (web-based, computer, mobile app, etc) | Occupational setting  (Industry) | Regulatorily support | Reliability | Evaluation approach (validation approach) | Additional findings related to the study/tool |
| --- | --- | --- | --- | --- | --- | --- | --- | --- | --- | --- | --- | --- | --- |
|  |  |  |  |  |  |  |  |  |  |  |  |  |  |
|  |  |  |  |  |  |  |  |  |  |  |  |  |  |
|  |  |  |  |  |  |  |  |  |  |  |  |  |  |
|  |  |  |  |  |  |  |  |  |  |  |  |  |  |
|  |  |  |  |  |  |  |  |  |  |  |  |  |  |
|  |  |  |  |  |  |  |  |  |  |  |  |  |  |
